# β-Adrenergic Receptor-Dependent Alterations in Murine Cardiac Transcript Expression Are Differentially Regulated by Gefitinib *In Vivo*

**DOI:** 10.1371/journal.pone.0099195

**Published:** 2014-06-05

**Authors:** Jennifer A. Talarico, Rhonda L. Carter, Laurel A. Grisanti, Justine E. Yu, Ashley A. Repas, Douglas G. Tilley

**Affiliations:** 1 Center for Translational Medicine, Thomas Jefferson University, Philadelphia, Pennsylvania, United States of America; 2 Center for Translational Medicine, Temple University School of Medicine, Philadelphia, Pennsylvania, United States of America; 3 Department of Pharmacology, Temple University School of Medicine, Philadelphia, Pennsylvania, United States of America; Emory University, United States of America

## Abstract

β-adrenergic receptor (βAR)-mediated transactivation of epidermal growth factor receptor (EGFR) has been shown to promote cardioprotection in a mouse model of heart failure and we recently showed that this mechanism leads to enhanced cell survival in part via regulation of apoptotic transcript expression in isolated primary rat neonatal cardiomyocytes. Thus, we hypothesized that this process could regulate cardiac transcript expression in vivo. To comprehensively assess cardiac transcript alterations in response to acute βAR-dependent EGFR transactivation, we performed whole transcriptome analysis of hearts from C57BL/6 mice given i.p. injections of the βAR agonist isoproterenol in the presence or absence of the EGFR antagonist gefitinib for 1 hour. Total cardiac RNA from each treatment group underwent transcriptome analysis, revealing a substantial number of transcripts regulated by each treatment. Gefitinib alone significantly altered the expression of 405 transcripts, while isoproterenol either alone or in conjunction with gefitinib significantly altered 493 and 698 distinct transcripts, respectively. Further statistical analysis was performed, confirming 473 transcripts whose regulation by isoproterenol were significantly altered by gefitinib (isoproterenol-induced up/downregulation antagonized/promoted by gefinitib), including several known to be involved in the regulation of numerous processes including cell death and survival. Thus, βAR-dependent regulation of cardiac transcript expression in vivo can be modulated by the EGFR antagonist gefitinib.

## Introduction

β-adrenergic receptor (βAR) stimulation contributes to both the acute regulation of cardiac contractility and the development of cardiac dysfunction under conditions of chronic catecholamine stress via engagement of Gs protein-dependent signaling pathways [1], [2]. In contrast to maladaptive Gs protein-mediated effects, it has been demonstrated that Gs protein-*in*dependent βAR signaling through transactivation of the epidermal growth factor receptor (EGFR) acts to preserve cardiac function and promote survival under conditions of chronic catecholamine stress [3]. While the upstream molecular mechanisms controlling βAR-mediated EGFR transactivation are known [3], [4], the downstream events that relay survival signaling in vivo are not well-characterized.

Recently, we reported that differential subcellular activation of mediators of cell survival, including extracellular-regulated kinases 1/2 (ERK1/2), occur in response to acute βAR-mediated EGFR transactivation in the whole heart and in isolated cardiomyocytes, most substantially within the nucleus [5]. Further, βAR-mediated regulation of cardiomyocyte gene expression was demonstrated to be sensitive to EGFR inhibition [5]. While this was the first study to demonstrate βAR-dependent effects on cardiomyocyte gene expression through EGFR transactivation, its scope was limited. Therefore, in this study we sought to determine if βAR-mediated EGFR transactivation has the capacity to regulate global changes in cardiac transcript expression in vivo. To this end, we performed whole transcriptome analysis of total RNA isolated from left ventricular tissue following acute treatment of C57Bl6/J mice with the βAR agonist isoproterenol in the presence or absence of the EGFR antagonist gefitinib. Herein, we report that both agents induce significant alterations in cardiac transcript expression and identify distinct cohorts of βAR-dependent transcripts that are differentially sensitive to gefitinib.

## Methods

### Animals

Ethics statement: All mice (C57Bl6/J, male and female) were purchased from Jackson Labs, housed and underwent drug treatment according to animal care and use protocol #4091, approved by the Temple University Institutional Animal Care and Use Committee, wherein all efforts were made to minimize suffering. The mice were given filter-sterilized i.p. injections of either vehicle (0.1% DMSO [D 4540, Sigma, St. Louis, MO] in PBS) or gefitinib (Gef, 5 mg/kg [G-4408, LC Laboratories, Woburn, MA]) followed 10 min later by an additional i.p. injection of either vehicle (sterile phosphate buffered saline [PBS]) or isoproterenol (ISO, 1 mg/kg [I6504, Sigma, St. Louis, MO]). After either 10 or 60 min the mice were euthanized according to the approved protocol above, their hearts were excised and their left ventricular tissue isolated, snap frozen in liquid N_2_ and stored at −80°C until further analysis.

### Immunoblotting

Left ventricular samples were homogenized in lysis buffer containing 20 mM Tris (pH 7.4), 137 mM NaCl, 10% glycerol, 1 mM EDTA, 1% NP-40, 10 mM NaF (chemicals attained from Fisher Scientific, Pittsburgh, PA), 1X HALT protease inhibitor cocktail (78437, Thermo Scientific, Rockford, IL) and phosphatase inhibitor cocktail set IV (524628, Calbiochem, USA). Lysates were run on 10% SDS-PAGE gels, transferred to Immobilon-P^SQ^ polyvinylidene fluoride 0.2 µm pore size membranes (Millipore, Billerica, MA) and probed overnight for phosphorylated ERK1/2 (1∶3000, Cell Signaling Technology, USA) or total levels of ERK1/2 (1∶5000, Millipore). After washing with TBS-T, membranes were incubated at room temperature for 60 min with IRDye680 Donkey anti-rabbit IgG (H+L) at 1∶20,000 (LI-COR Biosciences). Bound antibody was detected using the LI-COR Biosciences Odyssey System (LI-COR Biosciences). P-ERK1/2 intensities were normalized to corresponding T-ERK1/2 intensities and statistical analysis was performed via One-way ANOVA with a Newman-Keuls post hoc test with p values indicated in figure legends.

### Reverse Transcription Quantitative PCR

Total RNA was isolated from left ventricular samples using a Qiagen RNeasy Fibrous Tissue Midi Kit with Proteinase K and DNAseI digestion according to manufacturer’s protocol (Qiagen, Valencia, CA). cDNA was synthesized from the total RNA using the High Capacity cDNA Reverse Transcription kit (Applied Biosystems), and reverse transcription quantitative PCR (RT-qPCR) was performed with SYBR Select Master Mix (Applied Biosystems) in triplicate for each sample using recommended primer sets at an annealing temperature of 60.0°C. Assay primers sets used were: Mm01300401_m1 (*Nr4a1*), Mm00441325_m1 (*Sema3f*), Mm00449032_m1 (*Thbs1*), Mm00442191_s1 (*Aplnr*), Mm00439129_m1 (*Gck*), Mm00442968_m1 (*Mb*), Mm00457513_m1 (*Tef*) and Mm99999915_g1 (*GAPDH*). RT-qPCR data was analyzed using Applied Biosystems Comparative CT Method (ΔΔCT), using glyceraldehyde 3-phosphate dehydrogenase (*GAPDH*) to normalize expression of genes of interest and calculate relative quantitation (RQ) values for each. Validation of transcriptome results were performed on total RNA isolated from six mouse hearts per treatment condition, including those used for transcriptome analysis described below, on genes with a copy #>1 and significant alterations in one variant only. Statistical analysis was performed on the ΔCT data for each gene by one-way ANOVA with neuman-kewls post hoc test for statistical significance (P values listed in figure legends) using Prism 5.0 (Graphpad) and histograms are presented as RQ ± RQmin and RQmax.

### Transcriptome Analysis

Total RNA from 4 hearts per treatment condition were combined and submitted for whole transcriptome analysis with the SOLiD 4 platform (Applied Biosystems, Foster City, CA) as a fee-for-service using the Cancer Genomics Shared Resource (CGSR) at the Kimmel Cancer Center (Philadelphia, PA). A detailed description of RNAseq fragment mapping to the mouse genome using Life Technologies/Applied Biosystems Bioscope software (V. 1.3), where 1 copy number ≈ 3 reads per kilobase of exon per million mapped reads (RPKM), and differential gene expression analysis using Cufflinks software by CGSR has been described previously [6]. P values attained in the differential expression analysis were adjusted for multiple testing using the Benjamini-Hockberg correction [7], yielding q values. Genes were considered significantly altered if q <0.05 and the absolute fold-change in expression was ≥1.5. Hierarchical clustering was performed using Gene Cluster 3.0 [8] and Java TreeView [9]. Functional annotation of data sets, network and upstream regulator analyses were performed using Ingenuity Pathway Analysis (Ingenuity Systems, Redwood City, CA). Significance of association between sets of genes and related molecular and cellular functions was assessed using the Fisher’s Exact Test with P values <0.05 indicating a statistically significant non-random association. Functional and transcriptional activation predictions were based on the molecular relationships in the data sets that yielded z-scores, with a z-score ≥2 indicating activation and a z-score ≤−2 indicating inhibition.

### Data Availability Statement

The raw unfiltered transcriptome data is available at Dryad (http://doi.org/10.5061/dryad.j18q1).

## Results

**Figure 1:**
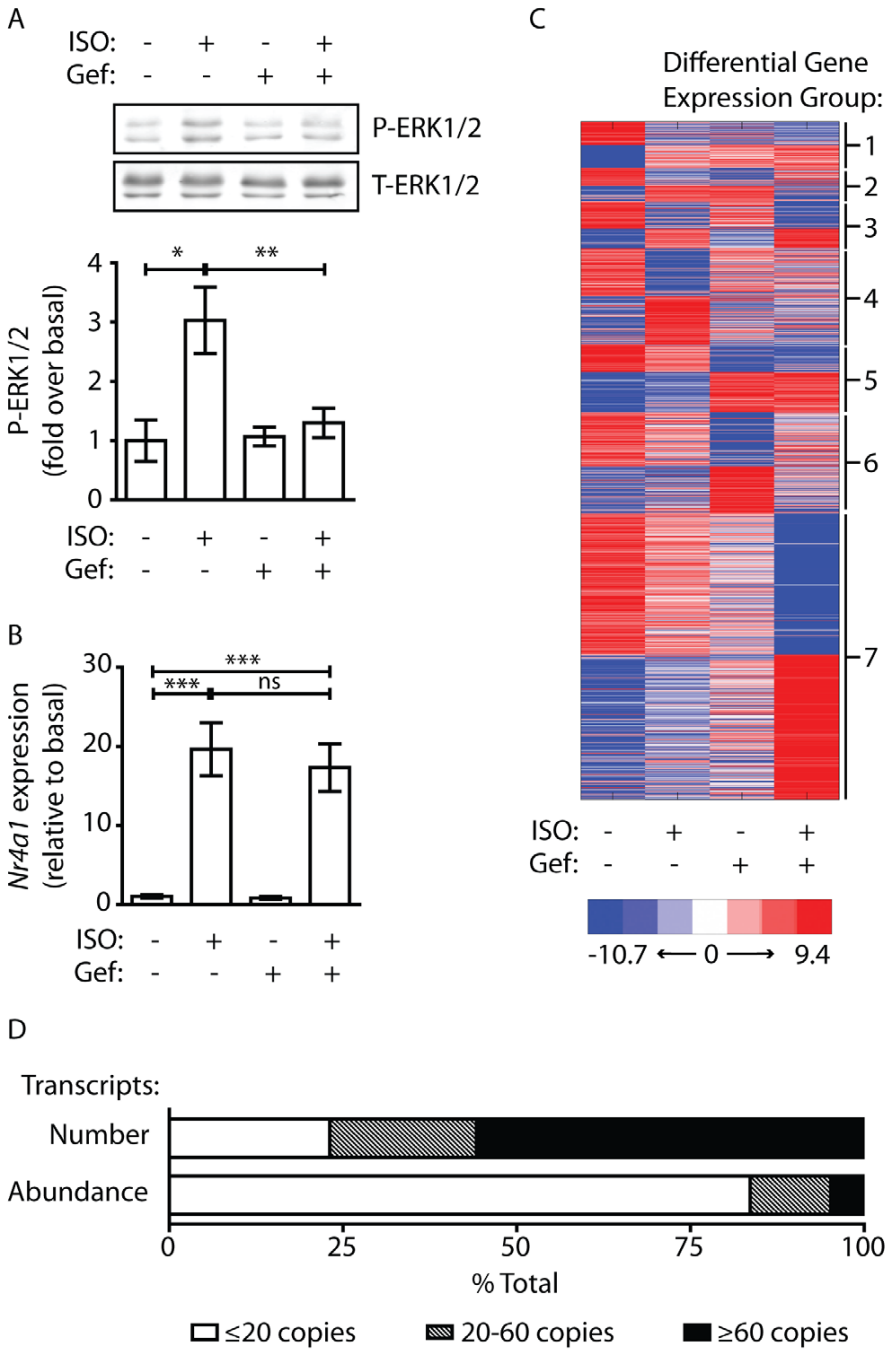
ISO and Gef differentially regulate acute changes in transcript expression in the mouse heart. A) C57BL/6 mice were given i.p. injections of ISO ± Gef for 10 min. Heart lysates underwent immunoblotting analysis for P-ERK1/2 and T-ERK1/2. ISO induced a 3-fold increase in P-ERK1/2 levels that was blocked by Gef pretreatment. Mean with SEM, ANOVA P value = 0.0123; *P<0.05, **P<0.01. B) RT-qPCR analysis of changes in the expression of the cAMP-regulated *Nr4a1* gene revealed a ∼20-fold increase in expression in response to ISO that was not blocked by Gef pretreatment. Data are presented as RQ with RQmin and RQmax as error bars; ΔCT ANOVA P value <0.0001; ***P<0.001, ns = not significant (P>0.05). C) Heatmap depicting significant (P<0.05) changes in cardiac transcript expression between vehicle-treated, Gef-treated, ISO-treated and Gef+ISO-treated mouse hearts as detected by transcriptome analysis. Differential transcript expression groups are listed to the right. D) Breakdown of cardiac transcripts into low, common and high abundance copies per cell categories with a comparison of the absolute number of transcripts (top bar) and relative abundance of transcripts (bottom bar).

Recently, we reported that acute βAR-mediated EGFR transactivation in rat neonatal cardiomyocytes reduces apoptotic signaling in part through the regulation of apoptotic gene expression [5]. To determine if βAR-mediated changes in gene expression are EGFR antagonist-sensitive at the organ level and to more broadly establish the ability of this mechanism to modulate acute cardiac transcript expression changes beyond regulation of only those involved in apoptosis, we performed whole transcriptome analysis from isolated mouse left ventricular RNA. Initially, C57BL/6 mice were treated with the βAR agonist isoproterenol (ISO) for 10 min in the presence or absence of the clinically used EGFR antagonist gefitinib (Gef). Using phosphorylated ERK1/2 (P-ERK1/2) as a standard readout for βAR-mediated EGFR transactivation [3]–[5], immunoblotting experiments were performed, confirming that treatment of the mice with Gef was sufficient to block ISO-induced phosphorylation of ERK1/2 in the whole heart (Fig. 1A).

Next, total RNA was isolated from the hearts of mice having received the same drug treatments, but harvested 1 hour after injection with ISO. To ensure that the treatments produced a predictable ISO-mediated gene expression response, prior to submitting the samples for transcriptome analysis, we performed RT-qPCR to measure changes in the relative expression of *Nr4a1. Nr4a1* expression is known to be induced rapidly through βAR-cAMP-PKA-CREB-dependent signaling [5], [10], [11] therefore its expression was expected to be enhanced by ISO treatment and unaffected by Gef treatment. Indeed, cardiac *Nr4a1* expression was significantly increased approximately 20-fold by ISO stimulation, while Gef pretreatment did not alter either basal or ISO-induced levels of *Nr4a1* (Fig. 1B).

Having confirmed a change in gene expression in response to pharmacological treatment, the samples were submitted for transcriptome analysis to assess global changes in cardiac transcript expression in response to ISO, Gef or Gef+ISO. Initial differential expression analysis filtered out all transcripts not significantly altered (P>0.05) within each treatment group versus the vehicle (Veh) control, revealing 3227 transcripts that were significantly altered by ISO or Gef alone or in combination (Fig. 1C). Classification into low abundance transcripts (≤20 copies/cell), common transcripts (between 20 and 60 copies/cell) and very high abundance transcripts (≥60 copies/cell) has been described previously by others where transcriptome analysis was shown to have a greater reliability for detecting significant changes in the low abundance transcript category compared to standard microarray analysis [12]. Sorting our transcripts into these classifications revealed that the majority fell into the low abundance category –83.6% of the total transcripts significantly altered by Gef, ISO or both. Only 11.6% and 4.8% of the altered transcripts could be classified into either the common or very high abundance groups, respectively, though the relative amount of these transcripts (21.1% and 55.8%) far exceeded those of low abundance transcripts detected (23.1%), even when excluding the extremely highly expressed *Snord* transcripts (Fig. 1D; unfiltered dataset at http://doi.org/10.5061/dryad.j18q1).

**Figure 2:**
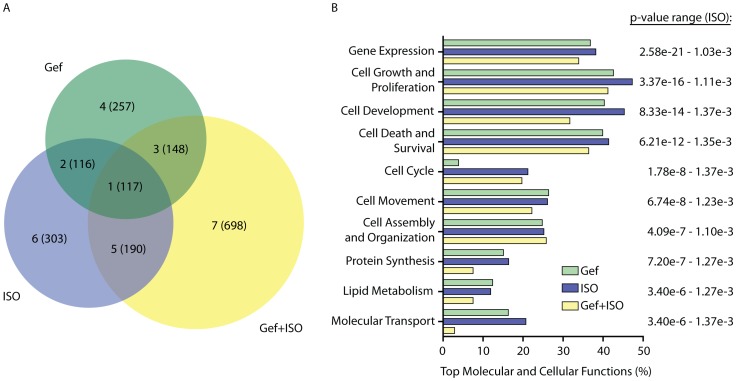
Classification of ISO and Gef-mediated effects on cardiac transcript expression. A) Biovenn-generated diagram depicting the number of cardiac transcripts significantly regulated by each treatment and adjusted for multiple testing (q <0.05). Numbers 1–7 correspond to the differential transcript expression groups in Fig. 1C, with numbers of transcripts in each group in parentheses. B) Ingenuity analysis revealed the ten most significantly associated molecular and cellular functions of the ISO-regulated transcripts (corresponding to differential expression groups 5 and 6 from the Biovenn diagram in A) along with the amount of Gef-regulated (groups 3 and 4) and Gef+ISO-regulated (group 7) transcripts in the same functional categories.

More stringent filtration of the data to exclude genes with q values >0.05 (false discovery rate-adjusted p value [7]) was performed leaving 1829 cardiac transcripts whose expression were significantly modified by either ISO (groups 5 and 6), Gef (groups 3 and 4) or Gef+ISO (group 7) or commonly by all treatments (groups 1 and 2) (Fig. 2A, Tables S1–S4). ISO (groups 5 and 6) induced alterations in the expression of 493 transcripts (259 up and 234 down) and ISO in the presence of Gef (group 7) altered another 698 transcripts (387 up and 311 down), while Gef (groups 3 and 4) altered 405 transcripts (193 up and 212 down). The 20 most highly altered transcripts by each treatment are listed in Table 1. Included in the list of ISO-sensitive genes is *Nr4a1*, with ∼36-fold (5.2 Log_2_-fold) increased expression over Veh-treated hearts, similar to the RT-qPCR data above. Indeed, the upstream regulator with the most highly significant association with the ISO-regulated gene cohort was determined through Ingenuity analysis to be CREB (p-value = 2.52E-15; z-score for activation = 4.157), confirming that transcriptome analysis of the cardiac RNA samples detected predicted and experimentally observed responses to ISO stimulation. Ingenuity analysis was performed to compare the relative quantity of genes regulated by each treatment within the ten most significant molecular and cellular functional categories attained for ISO-regulated genes, of which the most significantly associated functional category was gene expression (Fig. 2B). Although some variation in the impact on particular functions existed between the treatment groups, especially within the cell cycle and molecular transport categories, all three treatments were capable of inducing acute changes in cardiac transcript expression across several functional classifications.

**Table 1:** Fold-change summary of the 20 most highly altered cardiac transcripts within each treatment category.

Gef		ISO		Gef+ISO	
Transcript	Log_2_ Fold Δ	Transcript	Log_2_ Fold Δ	Transcript	Log_2_ Fold Δ
*Ddx17*	7.8	*Bclaf1*	7.7	*Repin1*	8.4
*Gtpbp2*	7.7	*Vegfa*	7.6	*Trim33*	7.7
*Arhgef1*	7.4	*Pias2*	6.4	*Ppm2c*	7.7
*Dusp27*	6.9	*Fosb*	6.4	*Dydc1*	5.9
*Grk6*	5.4	*Fos*	5.8	*Gm13103*	5.7
*Itgam*	5.1	*BC048671*	5.6	*Pias2*	4.7
*Magi1*	5.0	*Nr4a2*	5.5	*Lemd1*	4.6
*Ubp1*	4.1	*Nr4a3*	5.4	*Ret*	4.0
*Ccdc50*	4.0	*Nr4a1*	5.2	*Krtap8-1*	4.0
*Mgat1*	−4.3	*Lemd1*	5.0	*Alg12*	3.7
*Gatad2a*	−4.4	*Trpc4*	4.9	*Limk2*	−3.7
*Uspl1*	−4.4	*Atf3*	4.4	*Gm5506*	−4.0
*Sgk3*	−4.7	*Trim21*	−4.3	*Orc3l*	−4.1
*Kdm5d*	−5.1	*Ash2l*	−4.4	*Arhgap9*	−4.2
*Eif2s3y*	−5.1	*Atp11c*	−4.8	*Zswim3*	−4.2
*Uty*	−5.6	*Arid4b*	−5.3	*Nrp2*	−6.2
*Nrp2*	−6.2	*Acin1*	−6.4	*Cyth1*	−7.2
*Ddx3y*	−6.5	*Hmg20b*	−6.6	*Pde1c*	−8.0
*Zscan21*	−6.7	*Tmem183a*	−6.9	*Arhgap21*	−8.3
*Tapbp*	−10.5	*Tmem2*	−6.9	*Mb*	−8.7

**Figure 3:**
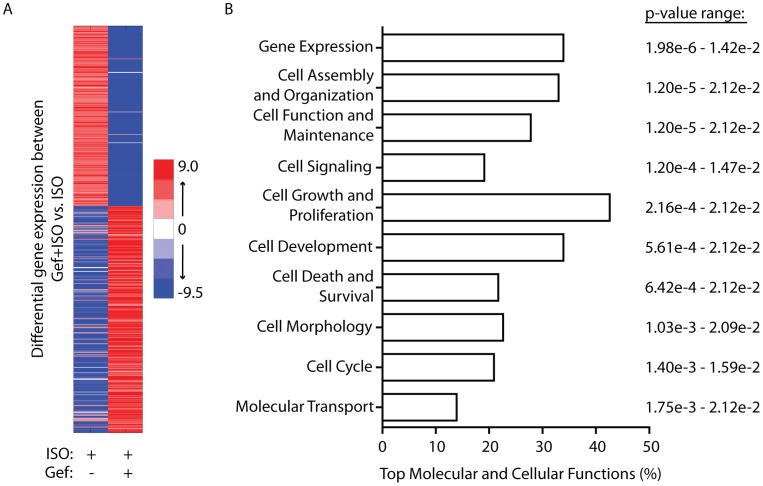
ISO-mediated regulation of cardiac transcript expression is sensitive to Gef. A) Heatmap depicting significant (P<0.05) changes in transcript expression between the ISO- and ISO+Gef-treated groups. B) Ingenuity analysis of the top molecular and cellular functions of ISO-regulated transcripts sensitive to Gef.

While each of the ISO and Gef+ISO treatment groups showed significant changes in expression of numerous genes versus vehicle, the data does not indicate how Gef treatment directly impacts ISO-induced cardiac gene expression per se. Thus, we performed differential expression analysis between the ISO and Gef+ISO groups (Fig. 3A), with subsequent statistical filtration as described above, revealing 473 cardiac transcripts (222 up and 251 down) that are not regulated by Gef alone, but whose ISO-dependent regulation is sensitive to Gef (Table S5), corresponding to approximately 40% of the genes significantly altered in the presence of ISO ± Gef (groups 5–7 in Fig. 2A). Consistent with a known role for βAR-mediated EGFR transactivation in promoting cardiac survival [3], Ingenuity analysis of the Gef-sensitive ISO-regulated transcripts predicted an increase in organismal death due to the directionality of the transcript expression changes with a z-score of 2.558 (p = 1.65e-5) and revealed a significant association of the altered transcripts with apoptosis activation (z-score of 2.286, p = 3.32e-3).

**Figure 4:**
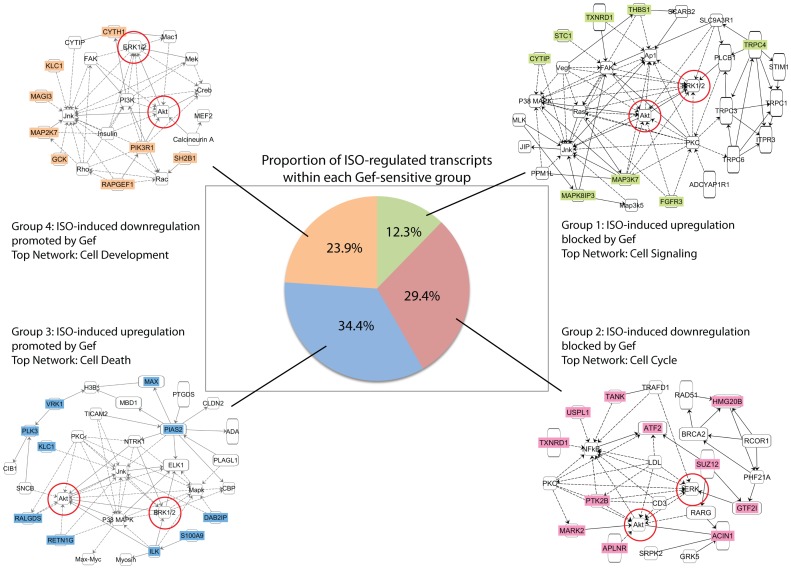
Network analyses of Gef-sensitive ISO-regulated transcript groups. Pie chart depicting the proportion of ISO-regulated cardiac transcripts (adjusted for multiple testing, q <0.05) that are sensitive to Gef, as well as their Gef-sensitivity classification. Ingenuity network analysis of each group’s top associated network function revealed a common inclusion of ERK1/2 and Akt (red circles) within each network (green = group 1, red = group 2, blue = group 3, orange = group 4). Solid lines indicate a direct relationship and dotted lines indicate an indirect relationship. Arrowheads indicate activation, perpendicular tags indicate inhibition and lack of either indicates an interaction.

Continued analysis of Gef-sensitive ISO-regulated transcripts focused on those with copy numbers >0 in all treatment groups and whose differential expression between the ISO and Gef+ISO could be confirmed by corresponding transcript expression changes in either the ISO vs Veh (Table S2) or Gef+ISO vs Veh (Table S3) groups, which narrowed the scope of the dataset to 163 transcripts. Ingenuity analysis of this cohort revealed a slightly different molecular and cellular function landscape than that observed by ISO alone. Although gene expression remained the most significantly associated category, alternate functions were associated with the Gef-sensitive ISO-regulated transcripts, such as cell function and maintenance, cell signaling and cell morphology (Fig. 3B). These transcripts could be further classified into 4 categories of Gef-sensitivity: 1) ISO-induced upregulation blocked by Gef, 2) ISO-induced downregulation blocked by Gef, 3) ISO-induced upregulation promoted by Gef, or 4) ISO-induced downregulation promoted by Gef (Fig. 4). Organization of these groups is provided in Table S6. Interestingly, a slight majority of the transcripts (58%) fall within groups 3 and 4, those whose ISO-dependent alteration in expression are only detected in the presence of Gef. Ingenuity network analysis of the distinct Gef-sensitivity groups was performed and although the top associated network functions of each group differed slightly, the core molecules within each network were remarkably similar with ERK1/2 and Akt, well-characterized mediators of EGFR signaling [5], repeatedly represented (Fig. 4).

**Figure 5:**
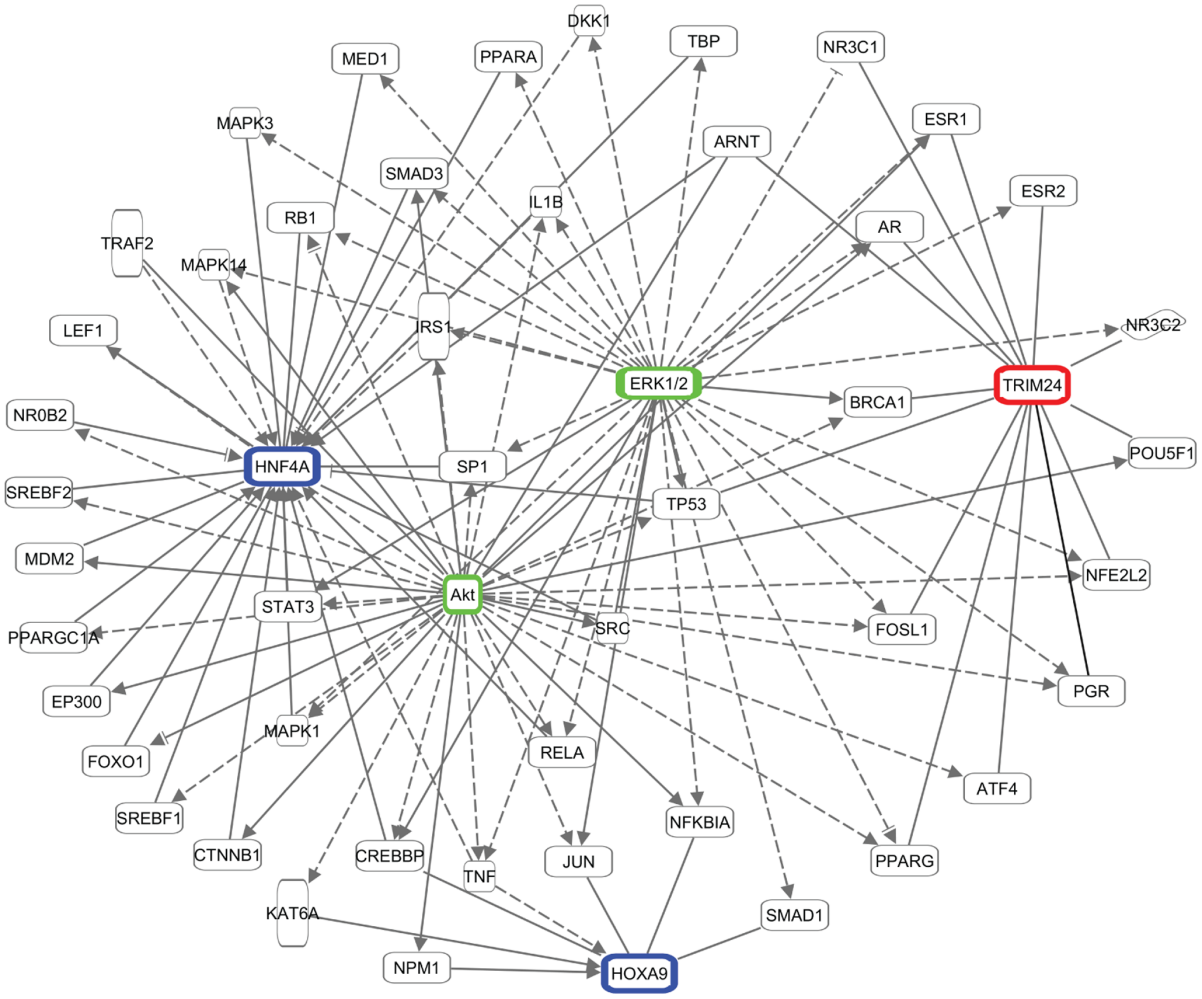
Predicted network relationships between ERK1/2 and Akt and transcription regulators in response to Gef-sensitive βAR signaling. ERK1/2 and Akt (green nodes) have been shown to directly modulate the activity and/or expression of numerous proteins that themselves have direct effects on the transcription regulators HOXA9 and HNF4A (blue nodes, predicted to be inhibited in the Gef+ISO condition) and TRIM24 (red node, predicted to be activated in the Gef+ISO condition). Solid lines indicate a direct relationship and dotted lines indicate an indirect relationship. Arrowheads indicate activation, perpendicular tags indicate inhibition and lack of either indicates an interaction.

To better understand the potential link between ERK1/2 and Akt activation via βAR-mediated EGFR transactivation and cardiac transcript regulation, we next performed upstream transcription regulator analysis of the Gef-sensitive ISO-regulated transcripts (Table S7). Of the 43 transcription regulators predicted to be involved in mediating changes in expression of these transcripts with p values <0.05, 3 also had absolute activation z-scores ≥2 based on the directionality of the transcripts altered by the Gef+ISO versus ISO cohorts: HOXA9 (p = 5.26e-3, z-score = −2.200) and HNF4A (p value = 1.38e-2, z-score = −2.000) were predicted to be inhibited under these conditions, while TRIM24 (p value = 3.82e-2, z-score = 2.236) was predicted to be activated. Network analysis of HOXA9, HNF4A and TRIM24 with ERK1/2 and Akt revealed that ERK1/2 and Akt are predicted to have indirect relationships with these transcription regulators via modulation of numerous other proteins that directly alter HOXA9, HNF4A and TRIM24 activity and/or expression (Fig. 5).

**Figure 6:**
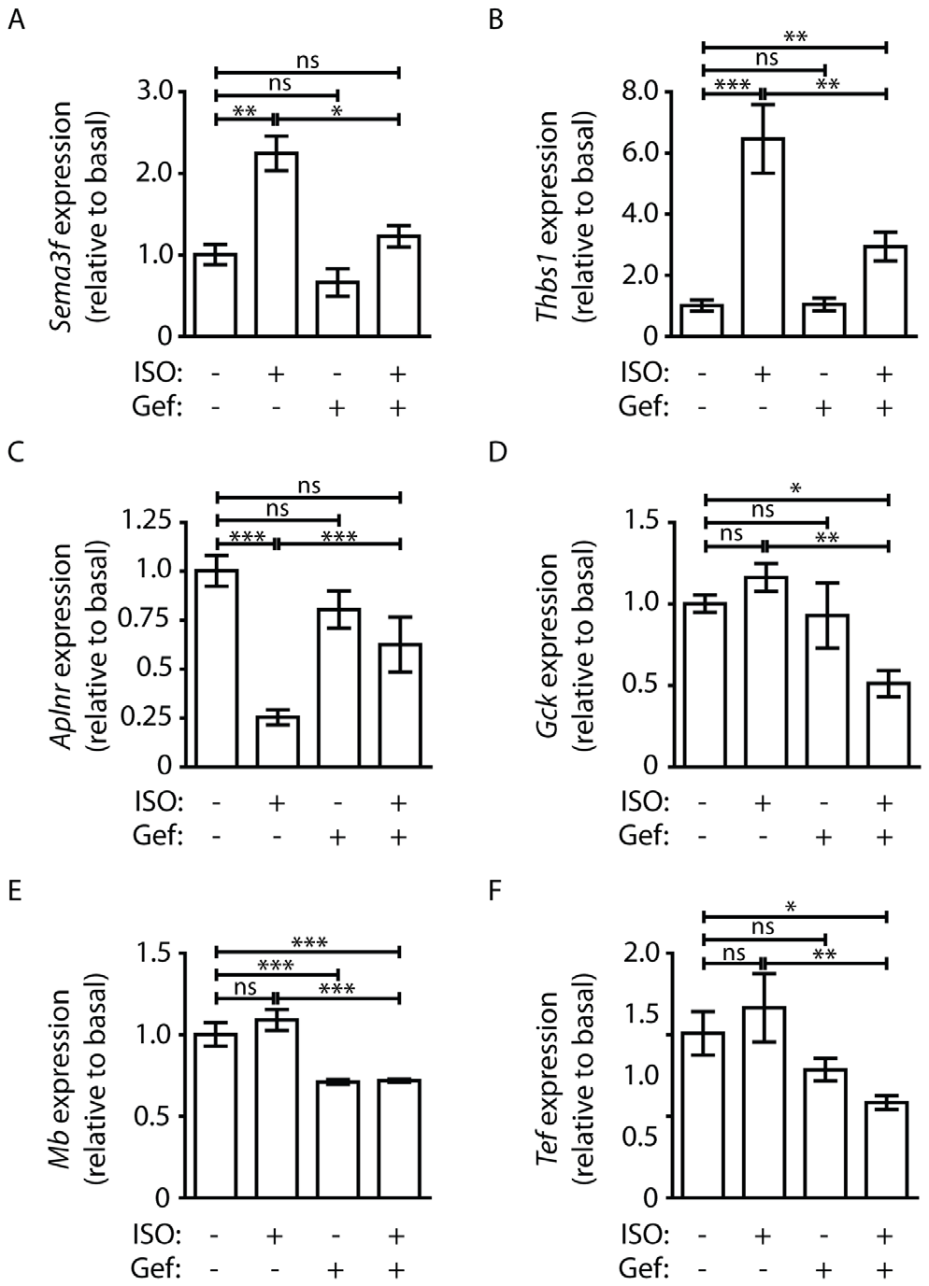
RT-qPCR validation of changes in expression of Gef-sensitive ISO-regulated transcripts. RT-qPCR analysis of changes in expression of transcriptss identified via transcriptome analysis: A) *Sema3f* (ΔCT ANOVA P value = 0.0002), B) *Thbs1* (ΔCT ANOVA P value <0.0001), C) *Aplnr* (ΔCT ANOVA P value <0.0001), D) *Gck* (ΔCT ANOVA P value = 0.0029), E) *Mb* (ΔCT ANOVA P value <0.0001) and F) *Tef* (ΔCT ANOVA P value = 0.006). Data are presented as RQ with RQmin and RQmax as error bars; *P<0.05, **P<0.01, ***P<0.001, ns = not significant (P>0.05).

To validate expression changes reported via transcriptome analysis, we performed RT-qPCR on several transcripts whose ISO-regulated expression was significantly altered by Gef. *Sema3f* expression was shown via transcriptome analysis to increase 2.6-fold in response to ISO, an effect completely blocked by Gef pretreatment and confirmed by RT-qPCR (Fig. 6A). *Thbs1* expression was also shown via transcriptome analysis to increase 7.4-fold in response to ISO stimulation, which was significantly decreased to only 2.1-fold over Veh by Gef pretreatment, observations that were closely mirrored via RT-qPCR (Fig. 6B). *Aplnr* was shown by transcriptome analysis to undergo a 5-fold decrease in expression in response to ISO, which was partially blocked by Gef. Via RT-qPCR analysis, a 4-fold ISO-induced reduction in *Apnlr* expression was also significantly antagonized by Gef pretreatment (Fig. 6C). Transcriptome results showed that ISO caused a decrease in the expression of *Gck*, *Mb* and *Tef* only in the presence of Gef, results that were confirmed via RT-qPCR (Fig. 6D–F) however Mb expression was also reduced to a similar extent by Gef alone, suggesting that this is an ISO-independent effect that was not detected in the transcriptome assay. Transcriptome versus RT-qPCR results are compared in Table 2, which, aside from *Mb*, indicate consistent transcript expression response patterns between analyses.

**Table 2:** Comparison of absolute fold-change differences in cardiac transcript expression via transcriptome and RT-qPCR analyses.

	ISO Vs. Veh		Gef+ISO Vs. Veh		Gef+ISO Vs. ISO	
Transcript	Transcriptome (Fold Δ)	RT-qPCR (Fold Δ)	Transcriptome (Fold Δ)	RT-qPCR (Fold Δ)	Transcriptome (Fold Δ)	RT-qPCR (Fold Δ)
*Sema3f*	2.59	2.23	1.05	1.22	−2.48	−1.83
*Thbs1*	7.4	6.36	2.08	2.90	−3.56	−2.20
*Aplnr*	−5.04	−3.94	−1.74	−1.60	2.90	2.45
*Gck*	1.10	1.16	−3.33	−1.96	−3.72	−2.27
*Mb*	1.70	1.09	−428.57	−1.39	−727.60	−1.52
*Tef*	1.18	1.15	−2.33	−1.73	−2.73	−2.00

## Discussion

**Figure 7:**
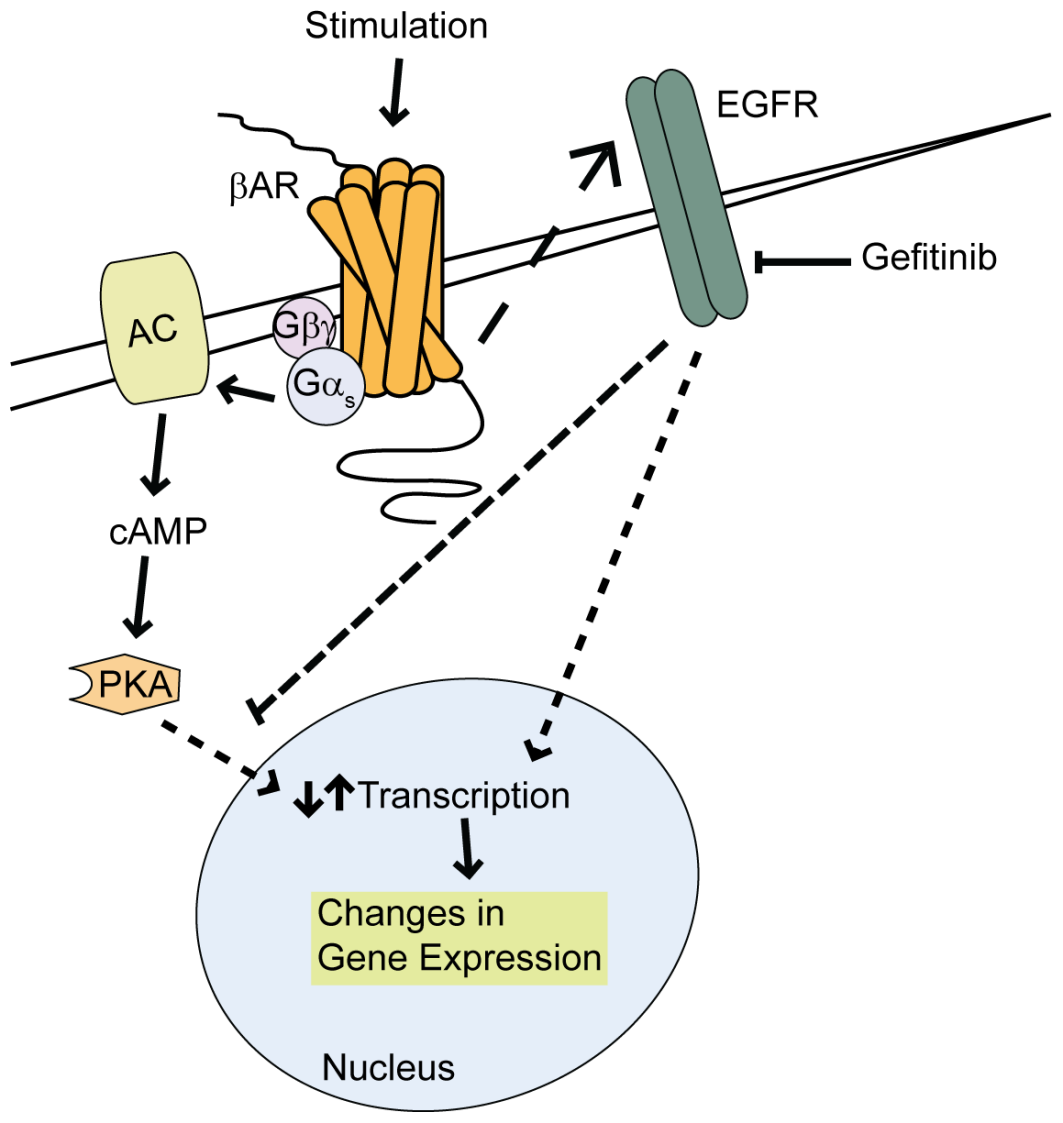
Impact of Gef on βAR-mediated regulation of cardiac transcript expression. Stimulation of βAR leads to activation of G protein signaling to induce changes in transcript expression via classical cAMP-PKA-mediated signaling. βAR stimulation also induces the transactivation of EGFR, which alters transcript expression directly, or indirectly by antagonizing the classical βAR-cAMP-PKA pathway, effects that are blocked with Gef treatment.

βAR-mediated transactivation of EGFR has been shown to relay cardiac survival in a mouse model of heart failure [3], but aside from ERK1/2 activation in a variety of cell types [4], [13], [14] and DNA synthesis in fibroblasts [15], the mechanisms by which protection are conferred via this process have not been well established. Recently, we reported that βAR-dependent EGFR transactivation increased activation of ERK1/2 and Akt within the nucleus of cardiomyocytes leading to acute regulation of apoptotic transcripts [5]. Since an alteration in cardiac transcripts could relay long-term effects on survival and function, we sought to more comprehensively assess the ability of EGFR transactivation to mediate acute changes in cardiac transcripts in vivo and define the relative contribution of this effect with respect to βAR-mediated regulation of cardiac transcript expression. Using transcriptome analysis, we have indeed shown for the first time that βAR stimulation induces a rapid alteration in cardiac transcript expression that is sensitive to the clinically-used EGFR antagonist gefitinib. Further, the sensitivity of βAR-dependent gene expression effects to Gef could be classified into four distinct groups: genes whose βAR-mediated upregulation is blocked (1), downregulation is blocked (2), upregulation is promoted (3) or downregulation is promoted (4) in the presence of Gef. There was an almost even distribution of the number of genes in groups 1 and 2 (∼42%) compared to groups 3 and 4 (∼58%), indicating that βAR-mediated EGFR transactivation not only acts to directly regulate cardiac transcript expression, but also to repress the effects of EGFR-*in*dependent βAR signaling on a subset of transcripts (Fig. 7).

Although a handful of reports have attributed the regulation of selected genes of interest to GPCR ligand (i.e. ET-1, AngII)-mediated EGFR transactivation-dependent mechanisms in various cardiomyocyte-like cell types [16]–[19], ours is the first study to comprehensively assess the early global alterations in cardiac transcript expression to βAR stimulation and establish a role for EGFR transactivation in this process in the whole heart in vivo. This approach has enabled us to identify several cardiac transcripts whose βAR-dependent regulation was not previously recognized, let alone the contribution of EGFR signaling in this response. Additionally, although Gef has previously been shown to alter gene expression, mainly in studies involving cancer cell lines [20]–[22], ours is the first report of Gef-induced changes in the expression of cardiac transcripts, including myoglobin (*Mb*). Myoglobin knockout mice were shown to be resistant to chronic ISO-induced cardiac stress and associated with alterations in the expression of genes involved in carbon metabolism, inhibition of apoptosis and muscle repair [23], thus a decrease in *Mb* expression in response to Gef treatment could be a compensatory pro-survival response. These acute alterations in cardiac transcripts imply that Gef treatment could influence changes in cardiac structure or function over time. However, Gef has not been associated with cardiotoxicity clinically [24], suggesting that acute alterations in gene expression mediated by Gef may not persist chronically and/or that such alterations do not overcome the threshold at which cardiotoxicity may be initiated.

Several cardiac transcripts were shown to be directly regulated via βAR-mediated EGFR transactivation, including *Sema3f, Thbs1* and *Aplnr*. *Sema3f* encodes semophorin 3F (Sema3F), a member of a large family of secreted and membrane-bound factors that influence cardiac innervation [25], inhibit angiogenesis [26], [27] and tumor invasion [28]. In context of the heart, semaphorins have been mainly studied for their role in development [29], [30]. With regard to injury in the adult heart, Sema3A was shown to be increased in response to chronic ISO infusion in rats [31] and to ameliorate electrical remodeling after myocardial infarction in rats [32]. The impact of Sema3f protein expression in the adult heart is not known, though its association with connexin 43 could be of importance [33]. *Thbs1* encodes thrombospondin-1 (TSP-1), a member of a matricellular protein family expressed at low levels in the heart normally but at higher levels in response to injury [34] and that have been shown to also act intracellularly to relay cardioprotection via reduced ER stress [35]. TSP-1 has been shown to also be antiangiogenic [36] and be confer protection in different models of cardiac injury including diabetes [37] and pressure overload [38]. Interestingly, a previous study showed that compared to wild-type mice *Thbs1* levels were elevated in mice with cardiac overexpression of β1AR, and augmented further by ablation of cAMP response element modulator (CREM), a transcription factor that, like CREB, can enhance cAMP-dependent transcription [39], [40]. Coupled with our results, this suggests that *Thbs1* expression may be enhanced in the absence of canonical Gs protein-dependent signaling via βAR-mediated regulation of EGFR, and could offer a cardioprotective advantage. In contrast to *Sema3f* and *Thbs1*, *Aplnr*, a gene that encodes for the apelin receptor (APJ), was shown to be reduced in response to ISO in a Gef-sensitive manner. Apelin/APJ signaling has been shown to be proangiogenic and provide beneficial outcomes in response to myocardial infarction in rats [41] whereas apelin levels are reduced in diseased murine and human hearts, increasing their susceptibility to myocardial ischemia-induced damage [42]. It is interesting to note that these validated transcripts from the Gef-sensitive groups 1 and 2 are associated with angiogenesis and cardioprotection, but the anti-angiogenic *Sema3F* and *Thbs1* transcripts were upregulated while the pro-angiogenic *Aplnr* was downregulated.

A slight majority of the Gef-sensitive ISO-regulated transcripts were those whose expression was not altered by ISO alone, but were altered when combined with Gef. This reveals a subset of cardiac transcripts, including *Gck* and *Tef,* that have the capacity to be regulated by βAR signaling, but their ISO-induced up- or downregulation are normally repressed through an EGFR-dependent mechanism. *Gck* encodes for glucokinase (or hexokinase IV), a glycolytic enzyme essential for glucose homeostasis via phosphorylation of glucose to promote glycolysis to increase intracellular energy, reduce stress-induced apoptosis and further enhance glucose uptake [43], [44]. The EGFR-dependent blockade of *Gck* downregulation by ISO fits with the pro-survival role of βAR-mediated EGFR transactivation in the heart, as a key step in glucose metabolism would be preserved. *Tef* encodes for thyrotroph embryonic factor (TEF), a transcriptional regulator that has been shown to be involved in the circadian control of cardiac transcription [45] and to increase the expression of the pro-apoptotic protein Bik, which increases oxidative stress-induced death in fibroblasts [46]. Although enhancement of pro-apoptotic protein expression would not relay a prosurvival EGFR signal, *Tef* deletion in mice has been associated with enhanced cardiac hypertrophy and left ventricular dysfunction [45], thus EGFR-dependent maintenance of *Tef* expression may ultimately promote survival.

The effects of chronic βAR stimulation (7–14 day ISO administration via osmotic minipumps) on global cardiac gene expression changes have been reported in recent years via microarray analysis, with a variable number of genes (∼200–1000) regulated in response to chronic ISO infusion [23], [47], [48]. βAR have been demonstrated to modulate the expression of transcripts, including those essential to the regulation of contractility, via several mechanisms including cAMP/CREB- and CAMKII-dependent regulation of transcription [1], [39]. Here, we have shown using transcriptome analysis that several hundred cardiac transcripts are altered in response to ISO (493; 259 up and 234 down) or to ISO in the presence of Gef (698; 387 up and 311 down) within an hour of stimulation in vivo. Further, we have demonstrated that inhibition of EGFR significantly impacts acute βAR-mediated regulation of cardiac transcript expression, a majority of which were associated with cell signaling, cell cycle, cell death and cell development networks, with ERK1/2 and Akt as commonly predicted network nodes that could contribute to cardioprotective signaling. However, the overall balance of cardiac transcript alterations and their ultimate contribution to βAR-mediated EGFR-dependent cardioprotective effects in chronic heart failure models remains to be tested.

### Study Limitations

While we have shown for the first time that βAR-mediated regulation of cardiac transcript expression is sensitive to EGFR antagonism in vivo, the use of a pharmacologic inhibitor does introduce the possibility of off-target effects contributing to the overall transcript alteration response observed. Of note, Gef has been shown to interact with and modulate the activities of the export pumps multidrug resistance protein 1 (MDR1) and breast cancer resistance protein (BCRP)[49]–[51] and such modulation could potentially alter cardiac transcript expression independently of EGFR. However, since neither ISO nor cAMP are reported substrates for these pumps and our differential analysis of Gef+ISO versus ISO cohorts removes a majority of transcripts regulated by Gef in the absence of ISO, we believe the remaining Gef-sensitive ISO-regulated transcript expression responses to be primarily reflective of inhibition of EGFR transactivation. Additionally, while ours is the first transcriptome analysis to characterize the impact of βAR-mediated EGFR-dependent stimulation on cardiac transcript regulation in adult hearts in vivo, our results are likely representative of changes occurring in several cell types, including cardiomyocytes, fibroblasts, endothelial cells and vascular smooth muscle cells. Since each of these cell types has been shown capable of inducing EGFR transactivation in response to GPCR stimulation [5], [52], [53], a more refined genetic approach would be useful in the future to definitively attribute cardiac transcript alterations specifically to EGFR within particular cardiac cell populations.

## Supporting Information

Table S1 **Gefitinib-sensitive changes in cardiac transcript expression.**
(XLSX)

Table S2 **Isoproterenol-sensitive changes in cardiac transcript expression.**
(XLSX)

Table S3 **Gefitinib+Isoproterenol-sensitive changes in cardiac transcript expression.**
(XLSX)

Table S4 **Common transcripts regulated independently by both Gefitinib and Isoproterenol.**
(XLSX)

Table S5 **Isoproterenol-induced changes in cardiac transcript expression that are sensitive to gefitinib.**
(XLSX)

Table S6 **Gefitinib sensitivity classification of isoproterenol-regulated cardiac transcripts.**
(XLSX)

Table S7 **Predicted upstream regulators of gefitinib-sensitive isoproterenol-regulated cardiac transcripts.**
(XLSX)
